# Drug-coated balloons versus drug-eluting stents for coronary de novo lesions in dialysis patients

**DOI:** 10.1007/s00380-022-02169-x

**Published:** 2022-09-01

**Authors:** Naohiro Funayama, Shingo Muratsubaki, Ryuta Ito, Toshiyuki Tobisawa, Takao Konishi

**Affiliations:** 1 Division of Cardiovascular Medicine, Hokkaido Cardiovascular Hospital, West 13, South 27, Chuou-ku, Sapporo, Hokkaido 064-8622 Japan; 2 Division of Cardiovascular Medicine, Hakodate Goryoukaku Hospital, 38-3, Goryoukaku-cho, Hakodate, Hokkaido 040-8611 Japan; 3 Division of Cardiovascular Medicine, Nagoya Kyoritsu Hospital, 1-172, Hokke, Nakagawa-ku, Nagoya, Aichi 454-0933 Japan; 4 Division of Cardiovascular Medicine, Asahikawa Red Cross Hospital, 1-1, Akebono, Asahikawa, Hokkaido 070-8530 Japan; 5 Division of Cardiovascular Medicine, Hokkaido University Hospital, West 5, North 14, Kita-ku, Sapporo, Hokkaido 060-8648 Japan

**Keywords:** Drug-coated balloon, Drug-eluting stent, De novo lesion, Dialysis patient

## Abstract

**Background:**

The aim of this study was to compare the efficacy of drug-coated balloon (DCB) angioplasty with drug-eluting stent (DES) angioplasty in the treatment of de novo coronary artery lesions in dialysis patients.

**Method:**

We retrospectively enrolled 400 consecutive dialysis patients with 464 coronary de novo lesions treated by DCB or DES from five participating institutions in Japan. The primary endpoint was target lesion revascularization (TLR) at 12 months. We performed serial coronary angiographic analysis.

**Results:**

There were no significant differences in the rate of TLR between the groups in either crude or propensity score-matched analysis (DES 14.1% vs. DCB 14.7%, *P* = 0.864, DES 12.1% vs. 12.1%, *P* = 1.00). Target lesion thrombosis was not observed in the DCB group; however, stent thrombosis was observed in 7 patients (2.2%) in the DES group. The rate of binary restenosis was similar in both groups (DES, 20.9% vs. DCB, 22.8%; *P* = 0.749). The late lumen loss at follow-up was significantly greater in the DES group than in the DCB group (0.61 ± 0.76 mm vs 0.22 ± 0.48 mm; *P* < 0.001). Late lumen enlargement was observed in 38.6% of patients in the DCB group.

**Conclusion:**

The efficacy of DCB angioplasty for de novo coronary artery lesions in dialysis patients was similar to that of DES angioplasty in the real world. Drug-coated balloon angioplasty can be an acceptable treatment for de novo coronary artery lesions in dialysis patients.

## Introduction

Percutaneous coronary intervention (PCI) with drug-eluting stents (DES) is an effective treatment for ischemic heart disease. Although DES is effective and safe in dialysis patients compared with bare metal stents (BMS) [1], these patients have higher restenosis rates than non-dialysis patients [2]. Patients on dialysis tend to have complex coronary artery lesions, such as massive calcification, which may lead to an increased risk of in-stent restenosis (ISR) [3]. Moreover, patients undergoing dialysis after PCI with new-generation DES have more adverse bleeding events [4]. Drug-coated balloon (DCB) angioplasty is a well-known, effective, and safe treatment for de novo lesions in coronary arteries [5]. The effectiveness of DCB angioplasty in the treatment of BMS-ISR in patients on hemodialysis (HD) has been demonstrated; however, DCB angioplasty in the treatment of DES ISR is less effective than repeat stenting with DES [6]. The effectiveness of DCB angioplasty for de novo coronary artery lesions in dialysis patients remains unclear. The aim of this study was to evaluate the efficacy of DCB angioplasty in the treatment of de novo coronary artery lesions in patients undergoing dialysis compared with DES angioplasty.

## Methods

### Study population and interventional procedures

This was a retrospective multicenter study to assess the efficacy of DCB angioplasty for the treatment of de novo coronary artery lesions in patients on dialysis. From June 2016 to September 2019, we retrospectively enrolled 400 consecutive dialysis patients with 464 coronary de novo lesions treated with DCB or DES from five participating institutions in Japan. We collected patient data, including those on coronary lesions and procedural characteristics, of all the patients. Eighty-eight patients with 99 lesions were treated with DCB (DCB group) and 312 patients with 365 lesions were treated with DES (DES group). All patients had established end-stage renal disease (ESRD) and had already been on maintenance dialysis before PCI. We performed all interventions according to standard techniques. The decision to perform PCI with DCB or DES was made by each operator. Patients in the DES group had new-generation drug-eluting stents, and those in the DCB group underwent PCI with SeQuent Please (B. Braun, Germany). The device and method for PCI, such as balloon and stent size, length, inflation pressure, use of intravascular imaging, and atherectomy devices, were left to the discretion of each operator. This study was approved by the Ethics Committee of each institution. The clinical follow-up information from each institution during the observation period was obtained via outpatient clinic visits, a review of the medical records, or by telephone.

### Angiographic analysis

We performed pre-procedure, post-procedure, and follow-up serial coronary angiograms. Angiographic follow-up was not mandatory and was performed either by physician request or upon findings indicative of myocardial ischemia. We performed Quantitative coronary analysis (QCA) of coronary angiographic data using the CAAS II Research System (Pie Medical Imaging, Maastricht, The Netherlands) at each angiogram. Reference vessel diameter (RVD), minimal lumen diameter (MLD), percentage diameter stenosis (%DS), and lesion length were measured. We calculated acute gain and late lumen loss (LLL) as post-procedure MLD minus pre-procedure MLD and post-procedure MLD minus follow-up MLD, respectively. Angiographic calcification was identified as readily apparent radiopacities within the vascular wall at the site of the stenosis and was classified as none/mild, moderate (radiopacities noted only during the cardiac cycle before contrast injection), and severe (radiopacities noted without cardiac motion before contrast injection) [7]. We defined binary restenosis as a stenosis diameter of at least 50% at follow-up and calcified lesions as lesions that were detected by coronary angiography. Late lumen enlargement was defined as lumen gain at the minimal lumen in the treated lesion (follow-up MLD > post-procedure MLD). QCA analysis was performed by experts at the Hokkaido Cardiovascular Hospital who were blinded to patient data.

### Endpoints

The primary clinical endpoint of this study was target lesion revascularization (TLR) within 12 months of follow-up. The secondary endpoints were cardiac death, myocardial infarction, target lesion thrombosis (TLT), and major adverse cardiac events (MACE). MACE was defined as repeat revascularization, cardiac death, or myocardial infarction. TLR was defined as any revascularization performed on the treated segment. Myocardial infarction was defined as an elevation of serum creatine kinase levels > 3 times the upper limit of the normal value. TLT was defined as an angiographic acute occlusion in a previously DCB- or DES-treated lesion.

### Statistical analysis

All statistical analyses were performed using the Statistical Package for the Social Sciences (SPSS) software (version 22.0; SPSS Inc. Chicago, Illinois, USA). We presented continuous variables as mean ± standard deviation and compared them between the two groups using paired Student’s *t*-test, Wilcoxon signed-rank test, or Mann–Whitney *U* test, as appropriate. We presented categorical variables as frequencies and percentages and compared them using the chi-square or Fisher’s exact test, as appropriate. A two-sided *p*-value of less than 0.05 was considered statistically significant.

To minimize selection bias, a propensity-score matching analysis was performed. Propensity score was calculated using logistic regression analysis which included variables such as gender, age, hypertension, diabetes mellitus, dyslipidemia, smoking history, etiology of renal failure, duration of dialysis, index presentation, and angiographic findings (lesion length, RVD, MLD, %DS) before the procedure. Kaplan–Meier analysis with log-rank test was used to express the cumulative incidence of TLR-free survival rate for comparison between the two groups.

## Results

### Baseline patient characteristics

Baseline patient characteristics are summarized in Table 1. All patients underwent maintenance dialysis. The mean duration from dialysis to PCI was 7.09 ± 5.77 years. The prevalence of diabetic nephropathy was > 60% in each group. A larger proportion of the patients had prior PCI and coronary artery bypass grafting (CABG) in the DCB group than in the DES group (58% vs. 40.7%; *P* = 0.004, 21.6% vs. 8.0%; *P* < 0.001, respectively).

**Table 1:** Baseline patient characteristics

	Overall (*n* = 400)	DES (*n* = 312)	DCB (*n* = 88)	*P*-value*
Age (years)	69.1 ± 10.1	69.3 ± 10.0	68.1 ± 10.3	0.344
Male	305 (76.3%)	237 (76.0%)	68 (77.3%)	0.888
Hypertension	350 (87.5%)	276 (88.5%)	74 (84.0%)	0.277
Diabetes mellitus	278 (69.5%)	213 (68.3%)	65 (73.8%)	0.360
Dyslipidemia	216 (54.0%)	172 (55.1%)	44 (50.0%)	0.400
Smoking history	136 (34.0%)	112 (35.9%)	24 (27.3%)	0.161
HD (versus PD)	380 (95.0%)	293 (93.9%)	87 (98.9%)	0.091
Etiology of renal failure				0.008
Diabetic nephropathy	260 (65.0%)	194 (62.2%)	66 (75.0%)	
Nephrosclerosis	47 (11.8%)	45 (14.4%)	2 (2.3%)	
Chronic glomerulonephritis	40 (10.0%)	29 (9.2%)	11 (12.5%)	
Other/unknown	53 (13.3%)	44 (14.1%)	9 (10.2%)	
Duration of dialysis (years)	7.09 ± 5.77	6.61 ± 5.24	8.39 ± 6.29	0.016
Index presentation				0.052
Stable angina	334 (83.5%)	254 (81.4%)	80 (90.9%)	
Unstable angina	42 (10.5%)	34 (10.9%)	8 (9.1%)	
NSTEMI	9 (2.3%)	9 (2.9%)	0 (0.0%)	
STEMI	15 (3.8%)	15 (4.8%)	0 (0.0%)	
Prior PCI	178 (44.5%)	127 (40.7%)	51 (58.0%)	0.004
Prior CABG	44 (11.0%)	25 (8.0%)	19 (21.6%)	< 0.001
Prior MI	61 (15.3%)	51 (16.3%)	10 (11.4%)	0.314
DAPT	390 (97.5%)	306 (98.1%)	84 (95.5%)	0.237
DAPT duration (months)	8.98 ± 3.73	9.19 ± 3.77	8.28 ± 3.55	0.046

Values are mean ± SD, *n* (%)

DES, drug-eluting stent; DCB, drug-coated balloon; HD, hemodialysis; PD, peritoneal dialysis; NSTEMI, Non- ST-segment elevation myocardial infarction; STEMI, ST-segment elevation myocardial infarction; PCI, percutaneous coronary intervention; CABG, cardiac artery bypass graft; DAPT, dual antiplatelet therapy

*P-value, DES vs. DCB

### Lesion and procedural characteristics

The lesion and procedural characteristics are summarized in Table 2. Before the procedure, the lesion length and RVD in the DES group were significantly longer and larger than those in the DCB group (18.4 ± 9.0 mm vs 14.3 ± 7.3 mm; *P* < 0.001, 2.65 ± 0.59 mm vs 2.42 ± 0.59 mm; *P* = 0.001, respectively). Angiographic analysis after the procedure showed that the results in the DES group were more acceptable, with larger MLD, lesser %DS, and greater acute gain than in the DCB group.

**Table 2:** Lesion and procedural characteristics

	Overall (*n* = 464)	DES (*n* = 365)	DCB (*n* = 99)	*P*-value*
Target vessels				< 0.001
LAD (Proximal, Mid, Distal)	198 (42.7%) (77, 107, 14)	166 (45.5%) (65, 92, 9)	32 (32.3%) (12, 15, 5)	
LCX (Proximal, Mid, Distal)	97 (20.9%) (32, 45, 20)	62 (17.0%) (21, 30,11)	35 (35.6%) (11, 15, 9)	
RCA (Proximal, Mid, Distal)	150 (32.3%) (55, 81, 14)	124 (34.0%) (47, 68, 9)	26 (26.3%) (8, 13, 5)	
SVG	4 (0.9%)	0 (0%)	4 (4.0%)	
LMCA	15 (3.2%)	13 (3.6%)	2 (2.0%)	
AHA Type B2/C	380 (81.9%)	290 (79.5%)	90 (90.9%)	< 0.001
Angiographic calcification				0.362
None/mild	124 (26.7%)	103 (28.2%)	21 (21.2%)	
Moderate	113 (24.4%)	88 (24.1%)	25 ((25.3%)	
Severe	227 (48.9%)	174 (47.7%)	53 (53.5%)	
Ostial lesion	53 (11.4%)	36 (9.9%)	17 (17.2%)	0.043
CTO	25 (5.4%)	21 (5.8%)	4 (4.0%)	0.622
Bifurcation	161 (34.7%)	147 (40.3%)	39 (39.4%)	0.207
Before procedure				
Lesion length (mm)	17.5 ± 8.8	18.4 ± 9.0	14.3 ± 7.3	< 0.001
RVD (mm)	2.60 ± 0.60	2.65 ± 0.59	2.42 ± 0.59	0.001
MLD (mm)	0.88 ± 0.52	0.90 ± 0.53	0.82 ± 0.49	0.211
%DS (%)	65.9 ± 19.0	65.9 ± 19.3	66.3 ± 18.1	0.846
After procedure				
RVD (mm)	2.91 ± 0.60	3.03 ± 0.54	2.50 ± 0.60	< 0.001
MLD (mm)	2.44 ± 0.60	2.62 ± 0.48	1.80 ± 0.57	< 0.001
%DS (%)	16.3 ± 11.1	13.2 ± 8.0	27.9 ± 13.2	< 0.001
Acute gain (mm)	1.56 ± 0.64	1.72 ± 0.60	0.97 ± 0.45	< 0.001
Device/lesion	1.07 ± 0.26	1.08 ± 0.27	1.03 ± 0.17	0.750
Device size				
Device diameter (mm)	2.91 ± 0.54	3.01 ± 0.51	2.54 ± 0.51	< 0.001
Device length (mm)	22.9 ± 8.1	23.7 ± 8.5	19.9 ± 5.0	< 0.001
Maximum inflation pressure (atm)	11.9 ± 3.2	12.8 ± 2.7	8.6 ± 2.5	< 0.001
Duration of inflation (s)		N/A	48.7 ± 11.6	N/A
Predilation performed	453 (97.6%)	359 (98.3%)	94 (94.9%)	0.062
Conventional balloon	131 (28.9%)	99 (27.6%)	32 (34.0%)	0.799
Scoring balloon	178 (39.3%)	145 (39.7%)	33 (35.1%)	
Cutting balloon	41 (9.1%)	32 (8.9%)	9 (9.6%)	
High pressure balloon	106 (23.4%)	83 (23.1%)	23 (24.5%)	
Balloon diameter (mm)	2.58 ± 0.49	2.62 ± 0.46	2.42 ± 0.53	< 0.001
Balloon length (mm)	13.3 ± 3.3	13.3 ± 3.2	13.2 ± 3.5	0.769
Maximum inflation pressure (atm)	13.8 ± 4.3	13.9 ± 4.1	13.5 ± 5.1	0.500
Rotablator	25 (5.4%)	19 (5.2%)	6 (6.1%)	0.802
Intracoronary imaging-guided PCI	418 (90.1%)	329 (90.1%)	89 (89.8%)	0.944
Postdilation performed		197 (53.9%)	N/A	N/A
Guiding catheter size (5/6/7/8 Fr)	21/358/80/5	17/275/69/4	4/83/11/1	0.315

Values are mean ± SD, *n* (%)

DES, drug-eluting stent; DCB, drug-coated balloon; LAD, left anterior descending; LCX, left circumflex; RCA, right coronary artery; CTO, chronic total occlusion; RVD, reference vessel diameter; MLD, minimal lumen diameter; %DS, percentage diameter stenosis; PCI, percutaneous coronary intervention

*P-value, DES vs. DCB

Before the procedure, the device size in the DCB group was significantly smaller than that in the DES group (2.54 ± 0.51 mm vs 3.01 ± 0.51 mm; *P* < 0.001). Type B2/C lesions were more than 90% in the DCB group. The type of pre-dilation balloon was not significantly different between the two groups. The maximum inflation pressure of the device was higher in the DES group than in the DCB group. Post-dilation was performed in more than half of the patients in the DES group.

### Endpoints

**Fig. 1:**
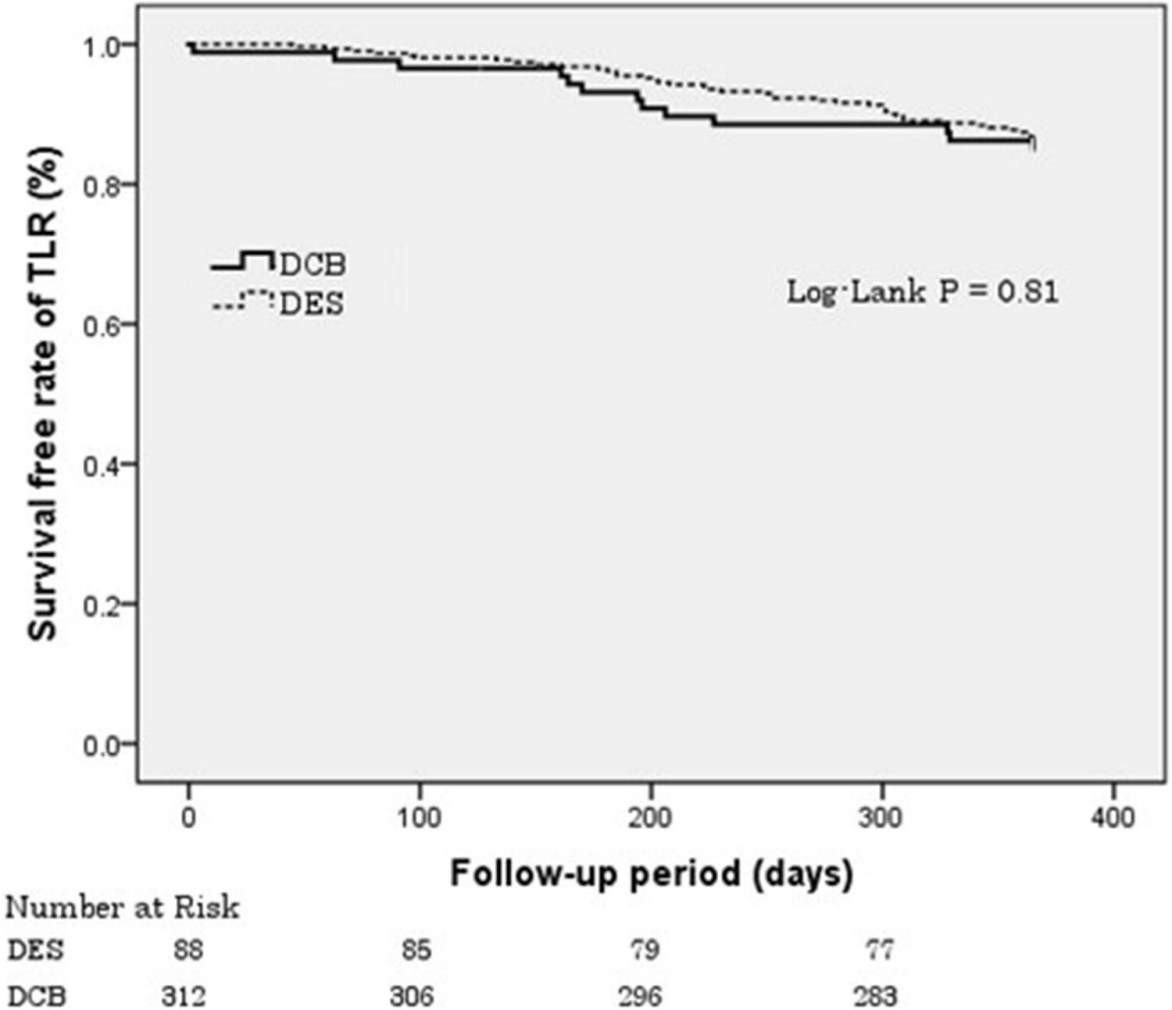
Survival-free rate of TLR. Kaplan–Meier estimated rates of freedom from TLR in dialysis patients treated with DCB and DES. TLR, target lesion revascularization; DCB, drug-coated balloon; DES, drug-eluting stent

The clinical outcomes are presented in Table 3. The overall rates of TLR and MACE were 14.3% and 18.3%, respectively. There were no significant differences in the rate of TLR, cardiac death, myocardial infarction, TLT, and MACE between the two groups. The Kaplan–Meier curve of the TLR-free survival rate is shown in Fig. 1. TLT was not observed in the DCB group; however, definite or probable stent thrombosis was observed in 7 patients (2.2%) in the DES group according to the Academic Research Consortium definition.

**Table 3:** Clinical outcomes at 12 months follow-up

	Overall (n = 400)	DES (n = 312)	DCB (n = 88)	P-value*
TLR	57 (14.3%)	44 (14.1%)	13 (14.7%)	0.864
Cardiac death	11 (2.8%)	9 (2.9%)	2 (2.3%)	0.757
Myocardial infarction	5 (1.3%)	5 (1.6%)	0 (0.0%)	0.590
TLT	7 (1.8%)	7 (2.2%)	0 (0.0%)	0.356
MACE	68 (17.0%)	53 (17.0%)	15 (17.0%)	0.990

Values are *n* (%)

DES, drug-eluting stent; DCB, drug-coated balloon; TLR, target lesion revascularization; TLT, target lesion thrombosis; MACE, major adverse cardiac event

*P-value, DES vs. DCB

### Post-propensity-score matching

After propensity-score matching, 66 matched pairs were obtained (Table 4). There was no significant difference between the groups in terms of baseline patient and lesion characteristics. There were no significant differences in clinical outcomes except for the rate of cardiac death between the DCB and DES groups (Table 5).

**Table 4:** Baseline patient, lesion characteristics post-propensity score matching

	DES (*n* = 66)	DCB (*n* = 66)	*P*-value*
Age (years)	69.6 ± 9.7	68.0 ± 10.3	0.389
Male	56 (84.8%)	53 (80.3%)	0.491
Hypertension	59 (89.4%)	55 (83.3%)	0.310
Diabetes mellitus	48 (72.7%)	45 (68.2%)	0.567
Dyslipidemia	31 (47.0%)	32 (48.4%)	0.862
Smoking history	22 (33.3%)	14 (21.2%)	0.118
HD (versus PD)	65 (98.5%)	65 (98.5%)	1.000
Etiology of renal failure			0.089
Diabetic nephropathy	45 (68.2%)	48 (72.7%)	
Nephrosclerosis	7 (10.6%)	1 (1.5%)	
Chronic glomerulonephritis	5 (7.6%)	10 (15.2%)	
Other/unknown	9 (13.6%)	7 (10.6%)	
Duration of dialysis (years)	6.77 ± 5.01	8.68 ± 6.79	0.068
Index presentation			0.121
Stable angina	55 (83.3%)	63 (95.5%)	
Unstable angina	8 (12.1%)	3 (4.5%)	
NSTEMI	2 (3.0%)	0 (0.0%)	
STEMI	1 (1.5%)	0 (0.0%)	
Prior PCI	25 (37.9%)	34 (51.5%)	0.115
Prior CABG	7 (10.6%)	12 (18.2%)	0.251
Prior MI	4 (6.1%)	6 (9.1%)	0.511
Target vessels			0.083
LAD (Proximal, Mid, Distal)	31 (47.0%) (13, 16, 2)	20 (30.3%) (9, 8, 3)	
LCX (Proximal, Mid, Distal)	14 (21.2%) (6, 7, 1)	22 (33.3%) (9, 9, 4)	
RCA (Proximal, Mid, Distal)	18 (27.3%) (10, 7, 1)	20 (30.3%) (6, 10, 4)	
SVG	0 (0.0%)	3 (4.5%)	
LMCA	3 (4.5%)	1 (1.5%)	
AHA Type B2/C	58 (87.9%)	60 (90.9%)	0.572
Angiographic calcification			0.736
None/mild	16 (24.2%)	20 (30.3%)	
Moderate	15 (22.7%)	14 (21.2%)	
Severe	35 (53.0%)	32 (48.5%)	
Ostial lesion	7 (58.3%)	12 (18.2%)	0.215
CTO	3 (4.5%)	1 (1.5%)	0.310
Bifurcation	16 (24.2%)	24 (36.4)	0.130
Before Procedure			
Lesion length (mm)	15.9 ± 7.9	15.6 ± 7.6	0.798
RVD (mm)	2.53 ± 0.52	2.56 ± 0.57	0.793
MLD (mm)	0.82 ± 0.47	0.89 ± 0.50	0.469
%DS (%)	67.7 ± 17.2	65.8 ± 16.8	0.534

Values are mean ± SD, *n* (%)

DES, drug-eluting stent; DCB, drug-coated balloon; HD, hemodialysis; PD, peritoneal dialysis; NSTEMI, Non- ST-segment elevation myocardial infarction; STEMI, ST-segment elevation myocardial infarction; PCI, percutaneous coronary intervention; CABG, cardiac artery bypass graft; DAPT, dual antiplatelet therapy; LAD, left anterior descending; LCX, left circumflex; RCA, right coronary artery; SVG, saphenous vein graft; LMCA, left main coronary artery; RVD, reference vessel diameter; MLD, minimal lumen diameter; %DS, percentage diameter stenosis

*P-value, DES vs. DCB

**Table 5:** Clinical outcomes at 12 months follow-up post-propensity score matching

	DES (*n* = 66)	DCB (*n* = 66)	*P*-value*
TLR	8 (12.1%)	8 (12.1%)	1.00
Cardiac death	4 (6.1%)	0 (0.0%)	0.042
Myocardial infarction	1 (1.5%)	0 (0.0%)	0.315
TLT	0 (0.0%)	0 (0.0%)	–
MACE	12 (18.2%)	8 (12.1%)	0.332

Values are mean ± SD, n (%)

DES, drug-eluting stent; DCB, drug-coated balloon; TLR, target lesion revascularization; TLT, target lesion thrombosis; MACE, major adverse cardiac event

*P-value, DES vs. DCB

### Angiographic outcomes

Angiographic outcomes are presented in Table 6. Angiographic follow-up was available for 57.8% (268/464) of all lesions. The mean follow-up duration was 244 ± 120 days.

**Table 6:** Angiographic outcomes

	Overall (*n* = 268)	DES (*n* = 211)	DCB (*n* = 57)	*P*-value*
Follow-up rate (%)	57.8% (268/464)	57.8% (211/365)	57.6% (57/99)	0.994
Before procedure				
Lesion length (mm)	17.8 ± 8.7	18.5 ± 8.9	14.8 ± 7.3	0.004
RVD (mm)	2.62 ± 0.62	2.68 ± 0.60	2.39 ± 0.64	0.002
MLD (mm)	0.85 ± 0.53	0.87 ± 0.53	0.77 ± 0.49	0.247
%DS (%)	67.7 ± 19.1	67.7 ± 19.3	67.6 ± 18.2	0.976
After procedure				
RVD (mm)	2.91 ± 0.60	3.02 ± 0.56	2.50 ± 0.61	< 0.001
MLD (mm)	2.43 ± 0.62	2.61 ± 0.49	1.73 ± 0.55	< 0.001
%DS (%)	16.8 ± 12.1	13.0 ± 8.3	31.0 ± 13.3	< 0.001
Acute gain (mm)	1.58 ± 0.67	1.75 ± 0.62	0.95 ± 0.45	< 0.001
Follow-up				
Duration of follow-up (days)	244 ± 120	250 ± 110	221 ± 148	0.10
RVD (mm)	2.79 ± 0.60	2.89 ± 0.56	2.42 ± 0.60	< 0.001
MLD (mm)	1.90 ± 0.84	2.01 ± 0.87	1.51 ± 0.61	< 0.001
%DS (%)	31.3 ± 26.8	29.8 ± 27.8	36.9 ± 21.8	0.74
Binary restenosis	57 (21.3%)	44 (20.9%)	13 (22.8%)	0.749
LLL (mm)	0.52 ± 0.72	0.61 ± 0.76	0.22 ± 0.48	< 0.001
LLE		N/A	22 (38.6%)	N/A

Values are mean ± SD, *n* (%)

RVD, reference vessel diameter; MLD, minimal lumen diameter; %DS, percentage diameter stenosis; LLL, late lumen loss; LLE, late lumen enlargement

*P-value, DES vs. DCB

Immediately after the procedure, %DS was significantly lower in the DES group than in the DCB group (13.0 ± 8.3% vs 31.0 ± 13.3%; *P* < 0.001), whereas there was no significant difference in %DS at follow-up. The rate of binary restenosis was similar in the DES and DCB groups (20.9% vs. 22.8%; *P* = 0.749). The late lumen loss (LLL) at follow-up was significantly greater in the DES group than in the DCB group (0.61 ± 0.76 mm vs 0.22 ± 0.48 mm; *P* < 0.001). Late luminal enlargement (LLE) was observed in 38.6% of patients in the DCB group.

## Discussion

The main findings of this retrospective study of dialysis patients are summarized as follows:

1. The efficacy and safety of DCB angioplasty for de novo coronary artery lesions in dialysis patients were similar to that of DES angioplasty; and
2. The late lumen loss (LLL) of DCB angioplasty was significantly lower than that of DES angioplasty.

The unique aspect of this study is that the outcomes of DCB angioplasty for de novo coronary lesions in dialysis patients were compared with those of DES angioplasty in the real world. To the best of our knowledge, there have been no previous studies which have examined the effects of DCB on de novo lesions in dialysis patients compared with DES in multicenter studies. DES angioplasty is well established as an effective intervention for coronary disease; however, in the era of new-generation DES, the poor clinical outcomes of dialysis patients are still available. Previous studies have shown that the TLR of DES angioplasty for dialysis patients is higher than that for non-dialysis patients [2, 6].

In the last few years, DCB angioplasty has emerged as an effective treatment for coronary artery disease, especially for small vessel lesions and in-stent restenosis lesions. However, DCB for ISR in dialysis patients had worse outcomes compared with non-dialysis patients [9]. In addition, very few studies have shown the efficacy of DCB for de novo coronary lesions in patients undergoing dialysis. DCB angioplasty for de novo lesions in HD patients had poorer outcomes than in non-HD patients [10].

The present study showed that the TLR rate after DES angioplasty was 14.1%. Some previous studies reported similar TLR rates (14.6% [11], 12.1% [12]). In this study, there was no significant difference in the TLR rate at 12 months between the DCB and DES groups. The study highlighted that the efficacy of DCB angioplasty for de novo coronary artery lesions in dialysis patients is approximately equivalent to that of DES angioplasty.

In contrast, there was no TLT in the DCB group. A previous study reported that treatment with DCB was associated with a similar risk of TLR and a lower risk of TLT compared with that with new-generation DES in non-dialysis patients [13]. Our study showed that the rate of stent thrombosis in the DES group was 2.1%. Previous studies reported similar rates of stent thrombosis in dialysis patients (2.0% [14], 1.3% [15]). Dialysis patients frequently have complex lesions, such as heavily calcified lesions, leading to suboptimal stent expansion. This may result in a high rate of restenosis and stent thrombosis. Konishi et al. [8, 16] reported that from the point of systemic problems, HD was associated with a high residual platelet reactivity, which may contribute to thrombus formation and MACE after DES implantation in patients undergoing HD. In addition, the potential disadvantage of low response to thienopyridine was observed in HD patients [17]. Furthermore, a previous study reported that dialysis patients who underwent PCI with DES implantation showed more adverse bleeding events compared with non-dialysis patients [4]. In another study, prolonged dual antiplatelet therapy (DAPT) in dialysis patients after DES implantation reduced MACE without significantly increasing major bleeding [18]; thus, the duration of DAPT after DES implantation in dialysis patients is uncertain. Although, in this study, there was no significant difference between DCB and DES in TLT, DCB might avoid TLT and may not require a long duration of DAPT because of the absence of a scaffold.

The binary restenosis and in-stent LLL in this study were 0.61 ± 0.7 mm, similar to that in previous studies (17.1%, 0.41 ± 0.71 mm [12] and 18.2%, 0.59 ± 0.78 mm [14]). In contrast, the LLL of the DCB group in this study was 0.22 ± 0.48 mm. Although this value was unfavorable compared with those of DCB angioplasty for non-dialysis patients in previous studies (0.08 ± 0.38 mm [19], 0.01 ± 0.31 mm [20] at 6 months), this study demonstrated that LLL was significantly lower in the DCB group compared with the DES group. There were no significant differences in the rates of binary restenosis and %DS at follow-up between DCB and DES angioplasty. The specific advantage of DCB treatment for de novo coronary lesions is the increase in lumen area in the chronic phase, referred to as LLE. LLE is generally observed in more than half of de novo coronary lesions treated with DCB [21]; however, in the present study, LLE was observed in 38.6% of the patients. The author previously reported that the mechanism of LLE has increased vessel and lumen area and decreased plaque area in treated de novo lesions in non-dialysis patients after DCB angioplasty [22]. A large proportion of dialysis patients in the DCB group had calcified lesions (76.8%). Although the calcified plaque was not a strong negative predictor of LLE [23], the lesions of dialysis patients have a relatively large amount of calcified plaque, leading to poor reduction of plaque area. Furthermore, there is a potential risk, including easy delamination of the drug on the surface of DCB when delivered through a proximal calcified segment in dialysis patients.

### Limitations

The present study had some limitations. First, we could not exclude the possibility of patient selection bias because this was a non-randomized, retrospective observational study. Furthermore, although propensity match score analysis was performed, the confounding factors might not be adjusted sufficiently. We enrolled dialysis patients who underwent PCI with DES or DCB, excluding other treatments such as plain old balloon angioplasty and CABG. Furthermore, there could be lesion selection bias due to PCI operator description. DCB angioplasty tends to be used in lesions with optimal preparation results, whereas DES angioplasty is used in lesions with potential issues such as acute recoil and severe dissection. To confirm the findings of this study, further randomized prospective studies in a large group of dialysis patients are needed. Second, as angiogram at follow-up was not mandatory, the rate of angiographic follow-up was approximately 60%. The data might be insufficient and silent ischemia could have been underestimated, but serial angiographic analysis of the lesions after DCB treatment in dialysis patients is valuable. Third, we were unable to describe the impact of calcified nodules due to a lack of an intracoronary imaging database. Dialysis patients often have calcified nodules associated with a worse prognosis.

## Conclusion

The efficacy and safety of DCB angioplasty for de novo coronary lesions in dialysis patients were similar to those of DES angioplasty in the real world. DCB angioplasty can be an acceptable treatment for de novo coronary lesions in dialysis patients.
